# Nanoparticles for improving biogas production and effluent biofertilizer

**DOI:** 10.1038/s41598-025-04131-z

**Published:** 2025-06-02

**Authors:** Taha Abdelfattah Mohammed Abdelwahab, Junting Pan, Ji-Qin Ni, Chunping Yang, Mahendra Kumar Mohanty, Elwan Ali Darwish, Samir Hafez Mohamed Desoky, Haiping Yang, Ahmed Elsayed Mahmoud Fodah

**Affiliations:** 1 Faculty of Agricultural Engineering, Al-Azhar University, Cairo, Egypt; 2 State Key Laboratory of Efficient Utilization of Arid and Semi-Arid Arable Land in Northern China, Institute of Agricultural Resources and Regional Planning, Chinese Academy of Agricultural Sciences, Beijing, 100081 China; 3 Department of Agricultural and Biological Engineering, Purdue University, West Lafayette, IN 47907 USA; 4 Academy of Environmental and Resource Sciences, School of Environmental Science and Engineering, Guangdong University of Petrochemical Technology, Maoming, 525000 Guangdong China; 5 Key Laboratory of Environmental Biology and Pollution Control (Hunan University), College of Environmental Science and Engineering, Ministry of Education, Hunan University, Changsha, 410082 Hunan China; 6 School of Environmental Science and Engineering, Hainan University, Haikou, 570228 Hainan China; 7 College of Agriculture Engineering and Technology, Odisha University of Agriculture & Technology, Bhubaneswar, Odisha 751003 India; 8 State Key Laboratory of Coal Combustion, School of Energy and Power Engineering, Huazhong University of Science and Technology, Wuhan, 430074 China

**Keywords:** Anaerobic digestion, Digestate, Kinetic model, Fertilizer, Nanoparticle mixture, Energy infrastructure, Renewable energy, Environmental sciences

## Abstract

The impact of nanoparticles (NP) mixtures (Fe + Ni + Co, Fe + Ni, Fe + Co, and Ni + Co) on the anaerobic digestion (AD) of cow manure was examined through kinetic modelling (Modified Gompertz, Logistic, and First-order kinetic) and experimental observations. The NPs mixture significantly enhanced biogas production rate and yield potential. The variation between predicted and actual biogas yields was smaller with the Modified Gompertz model (1.37% to 5.30%) and First-order kinetic model (0.04% to 3.83%) compared to the Logistic model (1.99% to 7.04%). Additionally, digestates containing NPs showed strong fertility properties (5.16% to 5.36%). The combined use of kinetic models and experiments offers an effective way to quantify the influence of NPs mixture on AD performance, aiding in industrial application.

## Introduction

Livestock contributes approximately 40% of the global agricultural product value and supports the livelihoods of nearly 1.3 billion people worldwide^1^. However, livestock farming also generates significant amount of waste, particularly from cow and poultry manure, with cow manure alone making up over 50% of total manure production in European^2^. Traditionally, cow manure is applied directly to land as fertilizer. This can cause environmental problems such as soil and water pollution, greenhouse gas emissions, and inefficient nutrient utilization^3^. Therefore, it is essential to develop methods to reduce the resource waste and environmental impacts associated with excess livestock manure^4^.

Anaerobic digestion (AD) has become a popular solution on dairy farms, offering a way to manage waste while producing biogas and harnessing the energy potential of cow manure^2,3^. Additionally, the digestate from AD can be used as a nutrient-rich fertilizer with lower greenhouse gas emission potential^5–7^.

The AD process consists of four stages, i.e., hydrolysis, acidogenesis, acetogenesis, and methanogenesis, each is carried out by different groups of bacteria, including hydrolytic, acidogenic, acetogenic, and methanogenic bacteria^8^. Many researchers have noted that hydrolysis is the rate-limiting step in the AD of lignocellulosic substrates like cow manure^9,10^. Consequently, several efforts have been made to boost microbial and enzymatic activity using trace elements such as iron (Fe), nickel (Ni), and cobalt (Co) in nanoparticle (NP) form to enhance methane production^11–15^.

Adequate levels of Fe, Ni, and Co are considered essential for the microbial communities involved in AD^16–18^. For Fe NPs, Wang et al. (2019)^19^ reported that 10 mg/g total solids (TS) of Fe NPs increased the number of microorganisms, bacteria, and archaea, as well as the activity of the F_420_ coenzyme, which is crucial for methanogen growth^20^. Abdelsalam et al. (2016)^11^ found that 20 mg/L of Fe NPs increased methane production by 59% compared to the control, while Abdelwahab et al. (2022b)^13^ observed a 118.8% increase with 30 mg/L of Fe NPs. For Ni NPs, Tsapekos et al. (2018)^21^ noted that 5 mg/Kg VS of Ni NPs increased methane production in the AD of sewage sludge by 10% compared to the control. Abdelsalam et al. (2017)^12^ found a 101% increase in methane production using 2 mg/L of Ni NPs. The finding aligns with Abdelwahab et al. (2023a)^14^, who reported a 71% improvement with the same concentration. Cobalt NPs have also been shown to promote acetogenesis during the startup of AD^15^, and Abdelsalam et al. (2017)^12^ observed a 186% increase in methane production with 1 mg/L of Co NPs compared to the control.

Mixtures of multiple NPs tend to be more beneficial for the AD process than individual NPs^22,23^. Zhang et al. (2015)^24^ found that the combination of Fe, Ni, and Co NPs increased biomass content in the AD system. Additionally, Zhang et al. (2011)^25^ showed that a mixture of Fe, Ni, Co, and Mo resulted in minimal accumulation of volatile fatty acids (VFAs), low soluble chemical oxygen demand (COD), and high solid degradation during the AD of piggery wastewater and food waste. Generally, the use of NPs (either single or combined) can enhance the AD process^22^. However, the interaction of Fe, Ni, and Co NPs on methane production and digestate quality is still not clear. Furthermore, an overestimation of NPs mixture addition may increase the operating costs. Therefore, gaining insight into the relation between using NPs mixture supplementation with minimal cost and methane production is crucial.

Kinetic analysis is essential for optimizing AD processes at a larger scale. Kinetic models help identify optimal variables, such as the lag phase and hydrolysis constant, while also assessing biogas production rates, AD potential, and the activation energy required for digestate pyrolysis^4,26,27^. Biogas production potential, lag phase, and hydrolysis rate constant parameters are key to understanding reaction mechanisms, optimizing process conditions, and predicting biogas yield, hydraulic retention time, and energy consumption in real-world applications. Despite the benefits of kinetic model fitting, the impact of NPs mixtures on AD, especially in terms of kinetic models and experimental data, has not been widely explored. Although NPs have been shown to improve biogas production in AD systems^11–15,28^, their influence on kinetic models for biogas production and digestate utilization remains understudied, distinguishing this research from prior work.

This work aimed to: (1) explore the effect of NPs mixtures on the biogas yield, TS and volatile solids (VS) removal rates, and pH variation; (2) to investigate the feasibility of digestate utilization by fertilizer analysis; (3) to predict the kinetic parameters (maximum biogas production rate and potential, lag phase, and hydrolysis constant) of biogas yield using the first-order kinetic model, logistic model, and modified Gompertz model. To the best of the authors’ knowledge, the effect of NPs mixture on the kinetic parameters of biogas yield and digestate is investigated for the first time.

## Materials and methods

### Experimental set-up

**Fig. 1:**
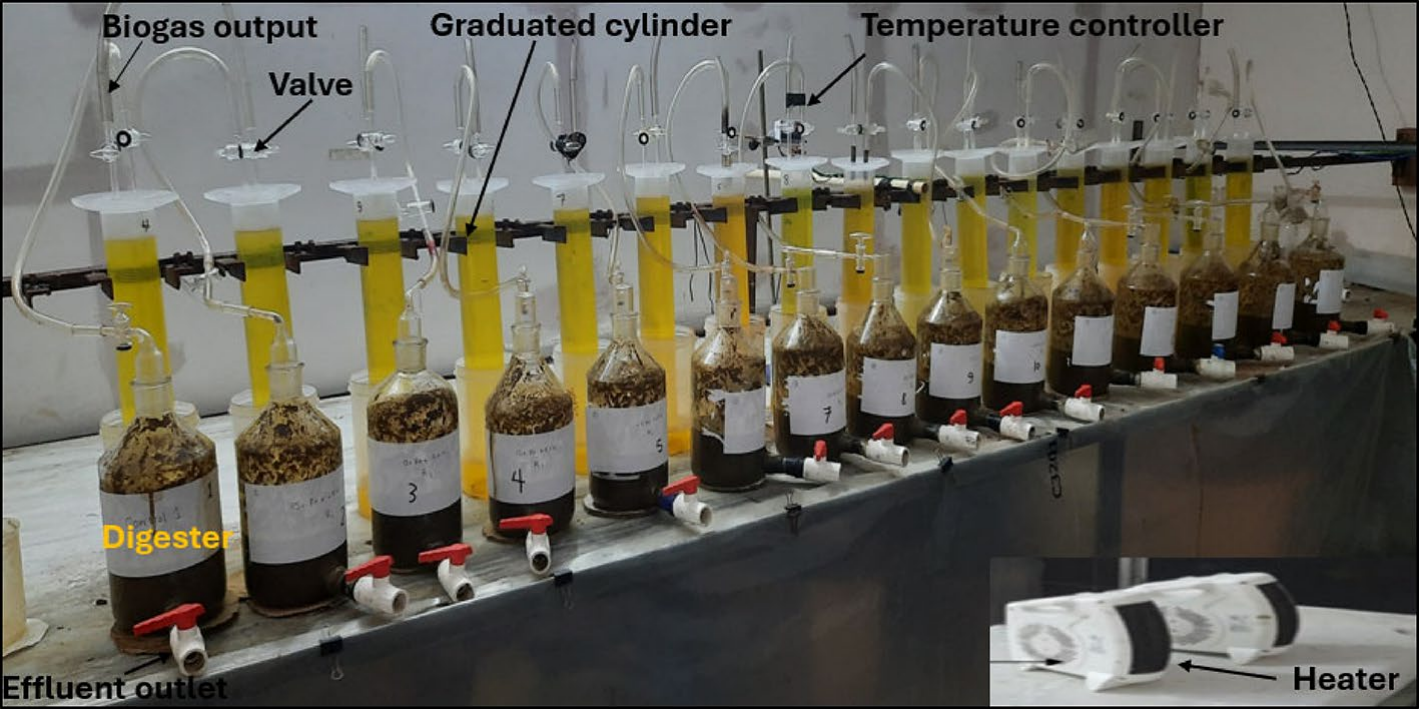
A visual diagram of the batch anaerobic laboratory scale digesters.

The batch mode was used with 15 laboratory-scale digesters in this study as shown in Fig. 1**.** Each digester consisted of a 1000-mL Borosil bottle with an effective working volume of 500 mL served as the digester and a 1000-mL clear plastic graduate cylinder served as the gas collecting unit. The graduated cylinder was placed upside down in another cylinder (2000 mL) filled with saturated sodium chloride (NaCl) solution. Both the digester and the gas collection cylinder were connected by rubber tubes. The experiments were conducted under mesophilic condition (33 ± 1℃). A complete explanation of the experimental devices can be found in our previous work^13–15^. Each digester was manually shaken three times a day, and its biogas volume was measured once a day.

### Substrate, inoculum, and nanoparticles

The digested cow dung was collected from a dairy farm in Benha City, Qalyubiyya, Egypt. Inoculant sludge was taken from a cow manure anaerobic digestion plant in Benha city, Qalyubiyya, Egypt. The detailed characteristic parameters of cow manure and inoculant sludge are summarized in Table 1. Cow manure 50 wt% and inoculant sludge 50 wt% were mixed well for each assay to make sure an optimal TS at 8–10% in the digester^4^. Nanoparticles Fe NPs, Ni NPs, Co NPs (99.9% metals basis) were obtained from Sigma scientific services Co. (Cairo, Egypt).

**Table 1:** Characteristics of cow manure and inoculant sludge.

Parameter	Cow manure	Inoculant sludge
MC (%)	90.87 ± 3.05	90.1 ± 3.00
TS (%)	9.13 ± 0.30	9.90 ± 0.32
VS (%)	76.13 ± 1.50	77.60 ± 1.60
AC (%)	23.87 ± 0.47	22.40 ± 0.46
VFAs (mg/L)	5100 ± 40	6000 ± 46
TA (mg/L CaCo_3_)	4000 ± 75	4600 ± 50
VFA/TA ratio	0.89 ± 0.01	1.2 ± 0.01
pH	7.10 ± 0.02	6.50 ± 0.01

MC, moisture content; TS, total solid; VS, volatile solids; AC, Ash content; VFAs, volatile fatty acids; TA, total alkalinity.

## Experimental design

Based on our previous work^13–15^, optimal concentrations of Fe NPs, Ni NPs, and Co NPs were selected at 30 mg/L, 2 mg/L, and 1 mg/L, respectively. The composition of different NPs mixture is presented in Table 2**.** The digesters in each group were triplicated. All digesters included 47.0 g dry (9.13 g VS) cow dung and 49.5 g dry (9.09 g VS) inoculant sludge. All batches were triplicated, and the results were repeatable.

**Table 2:** The concentration of different nanoparticle mixtures for each group.

Groups	Fe	Ni	Co
1	0 mg/L	0 mg/L	0 mg/L
2	30 mg/L	2 mg/L	1 mg/L
3	30 mg/L	2 mg/L	0 mg/L
4	30 mg/L	0 mg/L	1 mg/L
5	0 mg/L	2 mg/L	1 mg/L

### Analytical method

During the digestion period, the daily biogas production was determined using a simple water displacement method. Biogas production was expressed in unit of mL/g VS, which represented the volume of biogas when per gram of feedstock VS added to the digester. The APHA recommended standard procedures for determining TS and VS^29^. A digital pH meter (XL600, Germany) was used to measure the pH values before and after digestion. The contents of VFAs in each digester were determined using a titration method that combined the acidity (Method 2310B) and alkalinity (Method 2320B) titration methods^29^. The total levels of carbon, nitrogen, sulfide, and hydrogen (C, N, and S) were assessed using a micro elemental analyzer with simultaneous CHNS determination to evaluate the influence of NPs mixture on the chemical composition of the digestate (UNICUBE, Germany). After the experiments, Fourier transform infrared spectroscopy was used to characterise the organic content in the digestate samples (dry samples) (FTIR, Perkin Elmer Spectrum, version 10.4.3., USA). The total contents of phosphorus and potassium (TP and TK) were measured with an inductively coupled plasma optical emission spectrometer to examine the effect of NPs mixture on digestate fertility (ICP-OES, Perkin Elmer, Avio 200, USA).

### Kinetic model

Different process parameters such as biogas production potential, lag phase, and hydrolysis rate constant may be predicted by fitting the kinetic model to the biogas productin and digestate quality. This is crucial when assessing the beneficial effects of NPs mixture supplementation.

The modified Gompertz model (MGM) (Eq. (1)) depicts a standard sigmoidal curve that is used to explain microbial growth, and biogas production is considered a function of microbial growth in general^30^. The logistic model (LM) (Eq. (2)) assumes that the rate of biogas production is proportional to the quantity of biogas previously produced, the maximum production rate, and the biogas production potential, and that it can fit the fast growth in the beginning stage and eventually reach a stable level^26^. As a result, the MGM and LM were used in this study to calculate the biogas yield kinetic parameters. These two kinetic models were also used to determine the lag phase and maximum biogas production rate and evaluate the AD process, in addition to the biogas production potential.

1 $$P(t)={P}_{m}exp\left\{-exp\left[{R}_{m}\times e \times \left(\lambda -t\right)/{P}_{m}+1\right]\right\}$$

2 $$P\left(t\right)={P}_{m}/\left\{1+\text{exp}\left[4{R}_{m} \times \left(\lambda -t\right)/{(P}_{m}+2)\right]\right\}$$

where *P(t)* cumulative biogas production (mL/g VS) at time t (30 days); *P*_*m*_ is the maximum biogas potential production (mL/g VS); *R*_*m*_ is the maximum biogas production rate (mL/g VS/d); *λ* is the lag phase (d); *t* is the total digestion time (d);* e* is 2.71828.

The first-order kinetic model (FOM) is typically used to characterize the kinetics of AD fermentation process. Thus, the hydrolysis rate constant (*k*) of slurry can be fitted using Eq. (3)^31^:

3 $$P\left(t\right)= {P}_{m} .\left(1-{e}^{-kt}\right)$$

where *k* is the hydrolysis rate constant (d^-1^); *t* is time (d).

A second order Akaike Information Criterion (AIC) test was used to determine the model best matches the experimental results^26^. An AIC value can be positive or negative and the sign doesn’t have a meaning since it can be changed using different units to express data. Models were compared by evaluating the difference between the AIC values in which the model with the smallest AIC values was taken as the most likely to be correct. Equation (4) was used to calculate the AIC value^31^.

4 $$\text{AIC}=\left\{\begin{array}{c}Nln\frac{RSS+2n}{N}, when \frac{N}{n}\ge 40 \\ Nln\frac{RSS}{N}+2n+\frac{2n(n+1)}{N-n-1}, when \frac{N}{n} <40\end{array}\right.$$

where *N* number of points; *RSS* residual sum of square; *n* number of model parameters. The parameters for the models were determined by using SPSS IBM 20 software.

### Statistical analysis

Analysis of variance (ANOVA) test was performed on the cumulative biogas production with a significance level of 0.05 to analyze the statistical significance of the results. The statistical analyses were conducted using SPSS software, version 20 (IBM Co.).

## Results and discussion

### Effect of nanoparticle mixture on biogas production

#### Measured daily and cumulative biogas production

**Fig. 2:**
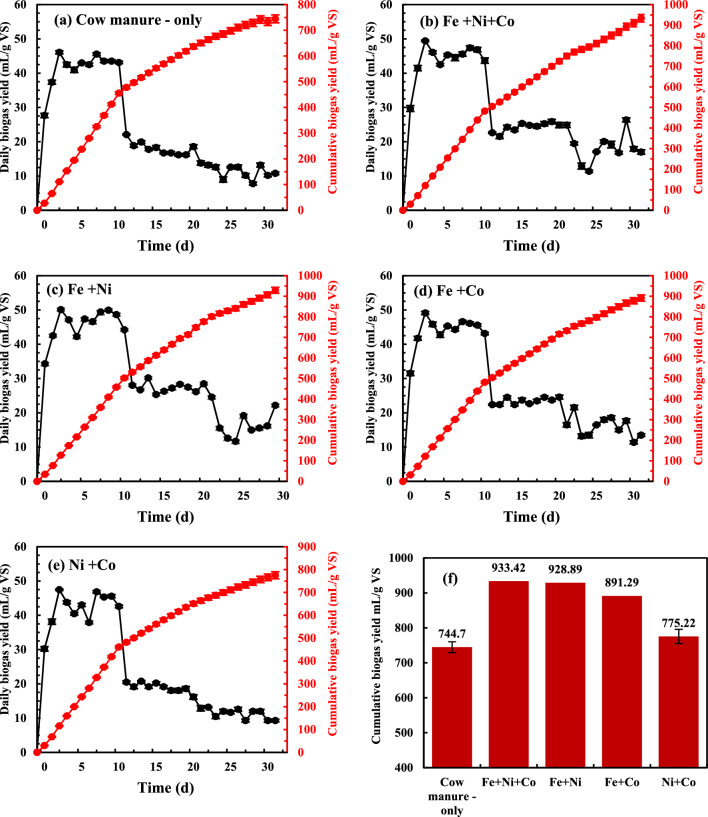
Daily and cumulative biogas produ**c**tion per g VS added in AD systems with (**a**) cow manure-only, (**b**) NPs of Fe + Ni + Co, (**c**) NPs of Fe + Ni, (**d**) NPs of Fe + Co, and (**e**) NPs of Ni + Co; and (**f**) comparison of cumulative biogas production of all five groups. Error bars in (**f**) are standard deviations.

The daily and cumulative biogas production over 32 days of AD are shown in Fig. 2. The trends in daily biogas yields with different additives showed similar patterns, with two peaks observed around day 3 and day 8. These peaks correspond to the degradation of carbohydrates and the breakdown of more complex organic materials such as crude protein and lignocelluloses^16,32^. Han et al. (2019)^33^ reported similar results, indicating that adding steel slag could boost biogas production and enhance digestate stability. In the current study, the two biogas production peaks for the Fe + Co + Ni group, measuring 49.43 and 47.36 mL/g VS, were not significantly different from the other groups: 50.10 and 49.82 mL/g VS for Fe + Ni, 49.12 and 46.08 mL/g VS for Fe + Co, 45.31 and 47.31 mL/g VS for Ni + Co, and 46.08 and 45.57 mL/g VS for cow manure only (p = 0.357).

As shown in Fig. 2 and Table 3, the Fe + Co + Ni group achieved the highest cumulative biogas yield at 933.42 mL/g VS, representing a 25.34% increase compared with cow manure alone. This was followed by the groups of Fe + Ni (928.89 mL/g VS, 24.73%, increase), Fe + Co (891.29 mL/g VS, 19.68%, increase), Ni + Co (775.22 mL/g VS, 4.10%, increase), and cow manure only (744.70 mL/g VS) (p < 0.05).

**Table 3:** Impact of NPs mixture on biogas yield kinetics.

Parameters	Group				
	Cow manure- only	Fe + Ni + Co	Fe + Ni	Fe + Co	Ni + Co
Measured final biogas yield (mL/g VS)-32 days	744.70 ± 13.60	933.42 ± 16.96	928.89 ± 14.45	891.29 ± 14.52	775.22 ± 14.26
**Modified Gompertz model (MGM)**					
*P*_*m*_ (mL/g VS)	742.23 ± 9.14	952.66 ± 21.20	985.21 ± 16.27	909.87 ± 17.10	767.65 ± 10.09
*R*_*m*_ (mL/g VS/d)	40.18 ± 1.30	39.51 ± 1.46	43.12 ± 1.28	39.88 ± 1.39	39.85 ± 1.30
λ (d)	0.221 ± 0.28	0.186 ± 0.39	0.186 ± 0.30	0.186 ± 0.36	0.275 ± 0.30
Predicted biogas yield (mL/g VS)-32 days	723.82 ± 13.21	886.37 ± 16.10	926.93 ± 14.00	857.99 ± 13.97	744.94 ± 13.69
Difference between measured and predicated biogas yield (%)	2.88	5.30	0.32	3.88	1.37
R^2^	0.994	0.992	0.995	0.993	0.994
AIC	188.12	210.53	199.89	204.24	190.35
**Logistic model (LM)**					
*P*_*m*_ (mL/g VS)	716.16 ± 11.27	896.98 ± 21.25	931.17 ± 18.02	862.78 ± 18.23	738.40 ± 12.09
*R*_*m*_ (mL/g VS/d)	38.80 ± 2.03	38.96 ± 2.02	42.62 ± 1.97	39.04 ± 1.99	38.57 ± 1.99
λ (d)	0.274 ± 0.52	0.242 ± 0.62	0.348 ± 0.53	0.270 ± 0.60	0.139 ± 0.53
Predicted biogas yield (mL/g VS)-32 days	710.75 ± 12.97	872.02 ± 15.84	910.70 ± 13.75	843.87 0 ± 13.74	731.46 ± 13.45
Difference between measured and predicated biogas yield (%)	4.77	7.04	1.99	5.61	5.98
R^2^	0.986	0.982	0.986	0.983	0.984
AIC	218.89	235.25	232.30	231.13	220.38
**First-order kinetic model (FOM)**					
*P*_*m*_ (mL/g VS)	895.12 ± 20.39	1277.34 ± 31.12	1317.43 ± 31.83	1175.51 ± 24.34	931.42 ± 19.72
*K*(d^-1^)	0.059 ± 0.003	0.040 ± 0.002	0.041 ± 0.002	0.044 0 ± 0.002	0.056 ± 0.003
Predicted biogas yield (mL/g VS)-32 days	758.05 ± 12.07	919.16 0 ± 13.70	964.89 ± 14.03	890.90 ± 13.66	778.48 ± 12.00
Difference between measured and predicated biogas yield (%)	1.79	1.55	3.87	0.043	0.42
R^2^	0.994	0.997	0.997	0.997	0.995
AIC	210.01	214.37	202.60	207.49	218.48

Previous studies have demonstrated that combining 500 mg/L nano zero-valent iron (nZVI) with 4 g/L of zeolite boosted biogas production by 130.87% compared to the control^34^. Similarly, Abdallah et al. (2019)^35^ found that using Ni-Ferrite nanoparticles at concentrations of 20, 70, and 130 mg/L increased biogas yields by 30.8%, 28.5%, and 17.9%, respectively, relative to cattle manure—only. Additionally, Karlsson et al. (2012)^36^ reported that adding a mixture of Fe, Co, and Ni chloride salts at concentrations of 500, 0.5, and 0.25 mg/L, respectively, to a semi-continuous biogas reactor enhanced biogas production by 23.91% compared to the control. These findings align with Farghali et al. (2019)^37^ who observed that mixtures of 20 mg/L Fe_2_O_3_ and 500 mg/L TiO_2_ nanoparticles, as well as 100 mg/L Fe_2_O_3_ and 500 mg/L TiO_2_, increased biogas production by 10.07% and 13.08%, respectively, compared to the control.

The use of NPs mixtures significantly increased biogas production compared to cow manure only, indicating that enhanced microbial activity in the digester accelerated the breakdown of organic matter. These results align with previous studies that demonstrated how supplementing methanogenic systems with micronutrients like Fe NPs, Co NPs, and Ni NPs improved key fermentation stages (hydrolysis and acidogenesis) and supported enzyme function^38–40^.

Furthermore, biogas production reached its highest level when Fe, Co, and Ni NPs were added together, consistent with the findings of^18^. Wang et al. (2021)^4^ also noted that digesters supplemented with a greater variety of trace elements, such as Fe, Ni, and Co, produced more biogas than those with fewer types.

The significant increase in biogas yield with the addition of NPs mixture can be attributed to the availability of additional micronutrients, which support microbial growth and enhance the activity of anaerobic bacteria. These results suggest that using a mixture of NPs could be an effective approach to improving AD performance.

#### Kinetic study of biogas production

Figure S1 (supplementary material) presents the fitting results of the three kinetic models (MGM, LM, and FOM) for biogas production, with Table 3 detailing the corresponding kinetic parameters. From Fig. S1a–c, it is evident that the MGM and FOM models align more closely with the measured data compared to the LM model. As shown in Table 3, the deviation between the fitted and measured biogas yields was higher for the LM model (1.99–7.04%) than for the MGM (0.32–5.30%) and FOM models (0.043–1.79%). Additionally, the R2 values for LM (0.982–0.986) were lower than those for MGM (0.992–0.995) and FOM (0.994–0.997). The results for Akaike Information Criterion (AIC) test for the MGM, FOM, and LM models are shown in Table 3. The MGM and FOM models have a lower AIC value than LM, indicating that they are better models to use in this case.

For the FOM model, the potential biogas production (*Pm*) increased with the addition of NPs mixtures, suggesting that incorporating NPs can enhance the biogas production potential in AD. Although *Pm* differed from the actual biogas yield, the R2 value remained within 0.994–0.997. The hydrolysis rate constant (*k*) for cow manure-only treatment, and the groups with Fe + Ni + Co, Fe + Ni, and Fe + Co NP mixtures, showed a reduction in the hydrolysis rate by 47.50%, 43.90%, and 34.09%, respectively. However, there was little difference in the *k* value between the Ni + Co group, which contained 2 mg/L of Ni NPs and 1 mg/L of Co NPs, and cow manure-only. These findings highlight the critical role of NPs mixture in affecting the hydrolysis rate.

For the MGM and LM models, biogas production potential (*Pm*) and the maximum biogas production rate (*Rm*) were improved by adding NPs mixture. For the Fe + Ni NP group, compared to cow manure-only, *Pm* increased by 32.73% for MGM and 7.31% for LM, while *Rm* increased by 30.02% for MGM and 9.80% for LM.

However, both models indicated that the NPs mixture had no significant impact on the lag phase (λ), a result consistent with Wang et al. (2021)^4^ and Zhang et al. (2019)^40^, who noted that trace element supplementation typically showed minimal short-term effects, with more pronounced benefits after extended operation.

These results suggest that the NPs mixture significantly enhanced biogas production potential and rates. Although FOM and MGM start from different theoretical assumptions, both models were successfully used to predict the impact of NP mixtures on biogas production, fitting well with the experimental data (R^2^ > 0.992). Thus, these models provide a reliable way to compare AD systems with and without NP mixtures.

However, when the substrate contains similar amounts of readily and poorly biodegradable components, two peaks in the cumulative biogas production curve may appear, reducing the fitting accuracy of classical models like FOM and MGM^41,42^. In such cases, classical kinetic models may need modification, or a more accurate model may need to be developed in the future to address these complexities.

### pH, removal rate of VFAs, TS, VS

The pH, VFAs, total alkalinity (TA), TS, and VS are all critical factors influencing the fermentation process^43,44^. The pH plays a crucial role in influencing the growth rate of microorganisms in AD systems^45,46^. In this study, the pH levels across all digesters ranged from 6.70 to 7.50 (Table 4), staying within the optimal range of 6.0 to 8.5^45,46^. This indicated that microbial activities within the AD systems were functioning properly.

**Table 4:** Parameter changes before and after AD.

Parameters	Group				
	Cow manure	Fe + Ni + Co	Fe + Ni	Fe + Co	Ni + Co
Initial pH	6.70 ± 0.03	6.70 ± 0.03	6.70 ± 0.03	6.70 ± 0.03	6.70 ± 0.03
Final pH	7.20 ± 0.01	7.50 ± 0.02	7.40 ± 0.03	7.30 ± 0.01	7.20 ± 0.02
Initial VFAs (mg/LCaCo_3_)	4875 ± 36	4875 ± 36	4875 ± 36	4875 ± 36	4875 ± 36
Final VFAs (mg/LCaCo_3_)	3200 ± 40	2800 ± 45	2820 ± 62	3000 ± 50	3050 ± 44
VFAs removal rate (%)	52.34 ± 0.7	74.10 ± 0.5	72.87 ± 0.6	62.50 ± 0.7	59.83 ± 0.8
Initial TS (%)	9.20 ± 0.11	9.20 ± 0.11	9.20 ± 0.11	9.20 ± 0.11	9.20 ± 0.11
Final TS	7.50 ± 0.08	6.50 ± 0.09	6.80 ± 0.09	7.00 ± 0.09	7.20 ± 0.08
TS removal rate (%)	22.66 ± 0.37	41.53 ± 0.22	35.29 ± 0.22	31.42 ± 0.22	27.77 ± 0.37
Initial VS	77.40 ± 1.90	77.40 ± 1.90	77.40 ± 1.90	77.40 ± 1.90	77.40 ± 1.90
Final VS	69.76 ± 1.71	64.21 ± 1.63	65.02 ± 1.65	66.21 ± 1.58	67.70 ± 1.75
VS removal rate (%)	10.95 ± 0.26	20.54 ± 0.42	19.04 ± 0.38	16.90 ± 0.55	± 0.10

The VFAs with short carboxylic chains (C2-C6) play a crucial role as intermediate products in the progression of AD^47,48^. During AD, VFAs are a key measure of the degradation of organic matter in substrates^49,50^. The varying rates of VFAs removal suggest that organic matter in the substrates was broken down by microorganisms to different extents, leading to biogas production^51,52^. As presented in Table 4, the group supplemented with Fe NPs, Ni NPs, and Co NPs achieved the highest VFAs removal rate of 74.10%, indicating more complete degradation and digestion of substrates compared to other experimental groups. This effect could be attributed to the Fe NPs, Ni NPs, and Co NPs additions, which supplied essential micronutrients for microbial growth.

The removal rates of TS and VS, which are key indicators for assessing the degradation of cow manure, were calculated and shown in Table 4. Compared to the cow manure-only group, the groups supplemented with Fe + Co + Ni, Fe + Ni, Fe + Co, and Ni + Co exhibited lower residual TS and VS after digestion. The Fe + Co + Ni group achieved the highest TS removal rate (41.53%) and VS removal rate (20.54%), which can be attributed to the combined addition of the NPs mixture. These findings are consistent with Wang et al. (2021)^4^, who reported that a trace element mixture of Fe, Ni, and Co improved TS and VS removal rate by 37.00% and 41.60%, respectively. However, the results of this study were lower than those reported by Farghail et al. (2019)^37^, who found that NPs mixture of 20 mg/L Fe_2_O_3_ and 500 mg/L TiO_2_ increased VS decomposition by 54.16% and 54.26%, respectively, compared to control conditions. This variation may be due to differences in the type and concentration of NPs mixture used in each study. Although the use of NPs mixture has shown promising results in enhancing biogas production, further research is needed to confirm its effectiveness for large-scale AD of cattle manure.

### Effects of NPs mixture on the characterization of organic material, chemical composition and fertility evaluation of digestate

The analysis of the organic material and chemical composition of the digestate is crucial for determining its quality^53^. The organic matter content and nutrient levels should support the soil microbial ecosystem and meet crop requirements when the digestate is applied as fertilizer in agricultural systems^54,55^.

#### Characterization of organic material and chemical composition of digestate

**Fig. 3:**
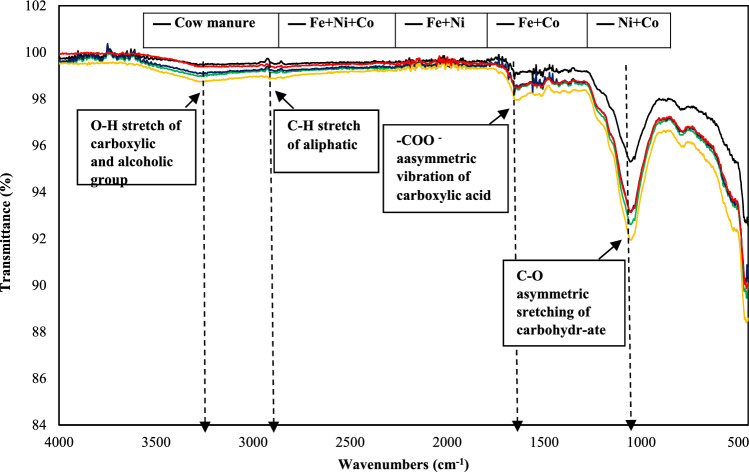
The FTIR spectra of the digestate with different NP mixtures.

The FTIR method was used to detect the characteristic vibrations of chemical bonds and functional groups. The FTIR spectrum of digestate, where the substrate was treated with a mixture of NPs, compared to untreated cattle manure, is shown in Fig. 3**.** The key absorption peaks identified, based on previous studies, include: (i) O – H stretching of carboxylic and alcoholic groups around 3450 cm^−1^, (ii) C–H stretching of aliphatic groups near 2900 cm^−1^, (iii) – COO − stretching of carboxylic acids around 1600 cm^−1^, and (iv) C – O stretching of carbohydrates near 1100 cm^−1^^55,56^.

In this study, the addition of various NPs mixture resulted in changes in both the intensity and shifts of these peaks compared to the untreated manure spectrum. Notably, a strong peak around 1100–950 cm^−1^, corresponding to C–O stretching of carbohydrates (Fig. 3), showed decreased intensity in the treated sample, indicating reduced carbohydrate content. This suggests that the NPs mixture may enhance methanogenic communities, promoting carbohydrate decomposition, VFA formation, and increased biogas production. Additionally, the peaks at 1600 cm^−1^ and 1509 cm^−1^ (N–H deformation vibration of amide II) were lower in the NPs treated groups, indicating enhanced organic matter degradation^57^. The FTIR analysis also revealed a slight shift to higher wave numbers due to NPs mixture addition. This may be attributed to interactions between soluble organic compounds and the NPs surface^58,59^.

#### Fertility evaluation of digestate

**Fig. 4:**
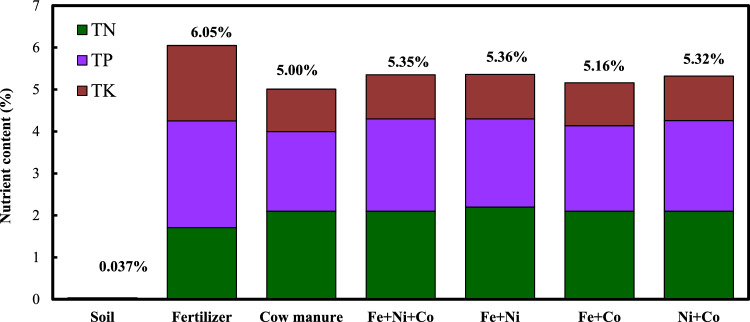
The NPK contents of soil, commercial bio-organic fertilizer and the digestate with different NPs mixture combinations.

Microbial degradation during AD increases the availability of nitrogen (N), phosphorus (P), and potassium (K) in organic matter, making digestates suitable for direct use as fertilizers or as components in commercial fertilizers^60,61^. The higher the concentration of these nutrients in digestates, the more effective they are as fertilizers. To assess the potential of digestates containing NPs as fertilizers, the total nitrogen (TN), total phosphorus (TP), and total potassium (TK) levels were measured for the Fe + Co + Ni, Fe + Ni, Fe + Co, and Ni + Co groups. As shown in Fig. 4, the NPK content was 5.35% for the Fe + Co + Ni group, 5.36% for the Fe + Ni group, 5.16% for the Fe + Co group, and 5.32% for the Ni + Co group. Since the NPK concentration for bioorganic fertilizers should exceed 5%, these digestates show promise as effective components of organic fertilizers.

Additionally, Fig. 4 compares the NPK content of these digestates with that of soil and commercial bioorganic fertilizers. The total nutrient content in the Fe + Co + Ni, Fe + Ni, Fe + Co, and Ni + Co groups was significantly higher than in soil, indicating their potential for soil enrichment. The nutrient levels of the digestates were also comparable to those in commercial bioorganic fertilizers, with the TN of the digestates exceeding that of the commercial products, making them particularly beneficial for nitrogen-deficient soils. However, the TP content in these digestates was lower than that of commercial fertilizers, a result consistent with other studies on digestates derived from cattle manure^40,62^. Further research is needed to develop phosphorus-rich additives for anaerobic digestion to improve the TP content in digestates.

### Cost analysis from nanoparticles input for biogas production

Analyze the costs was performed to investigate the impact of various NPs mixture on biogas yield. The cost calculation is based on the amount of CH_4_ produced (L) from one liter of substrate. As shown in Table 5, the highest net profit was attended with the group Ni + Co NPs, and was 0.51 USD over the cattle manure-only. Additionally, the groups of Fe + Ni + Co, Fi + Ni, and Fe + Co achieved net profit of 0.49, 0.38, 0.18 USD, respectively over the cattle manure-only. These results indicated that not only the supplementation of bio-digesters with NPs mixture of Fe, Ni, and Co boosted CH_4_ production, but also gained more profits from the commercial aspect of view. As a result, NPs mixtures may be a sustainable and cost-effective way to boost CH_4_ production from AD of cow manure.

**Table 5:** Cost analysis from NP mixture input for biogas production.

Item	Cattle manure-only	Fe + Ni + Co	Fe + Ni	Fe + Co	Ni + Co	Unit
1. Cumulative methane (L of substrate)	4.28	5.43	5.25	4.91	5.14	L
2. Income from methane production^a^	2.65	3.36	3.25	3.04	3.18	USD
3. Cost of NPs^b^	-	0.217	0.211	0.204	0.0196	USD
4. Net income (2–3)	2.65	3.14	3.039	2.83	3.16	USD
5. Net profit (4-net income of cattle manure-only)	0	0.49	0.38	0.18	0.51	USD

^a^Calculation of income from methane production was based on one L methane is equal to 0.620 USD (GlobalPetrolPrices.com).

^b^Calculation of NPs cost was passed on 1 g NPs (Fe, Ni, and Co NPs with the same price) is equal 6.6 USD (Sigma scientific services Co., Cairo, Egypt).

## Conclusions

The effect of a nanoparticles (NPs) mixture of Fe, Ni, and Co on the anaerobic digestion (AD) performance was investigated in a mesophilic AD process. The addition of NPs mixture significantly enhanced the AD of cow manure. The Fe + Co + Ni group achieved the highest volatile fatty acids (VFAs) removal rate (74.10%) and cumulative biogas yield (933.42 mL/g VS). Both the Modified Gompertz Model (MGM) and First-order Model (FOM) closely matched the experimental data, outperforming the Logistic Model (LM). The total solids (TS) removal rate of digestates in the NPs-treated groups also improved. The fertilizer potential of these digestates was comparable to that of commercial NPK fertilizers. Using a combination of kinetic models and experimental data can be an effective approach to evaluate the impact of NPs mixtures on AD performance.

## Supplementary Information

Supplementary Information.

## Data Availability

The datasets used and/or analysed during the current study available from the corresponding author on reasonable request.
