# Supplementary material for: Nanoparticles for improving biogas production and effluent biofertilizer
Source: Sci Rep. 2025 Jun 2;15:19233. doi: 10.1038/s41598-025-04131-z (PMC12127476; doi:10.1038/s41598-025-04131-z)
Supplement: Supplementary file 1 — Supplementary Information. [file 41598_2025_4131_MOESM1_ESM.docx]

**Fig. S1.** Cumulative biogas production from batch experiments (points) and kinetic model (solid curves) for the AD system with different groups of NPs: (a) MGM, (b) LM, and (c) FOM.
